# Revisiting the Women Workplace Culture Scale: Validation and Psychometric Properties of a Three-Factor Structure in an Iranian Study Sample

**DOI:** 10.3390/ejihpe10030065

**Published:** 2020-09-21

**Authors:** Ferdinando Toscano, Davide Giusino, Tayebe Rahimi Pordanjani

**Affiliations:** 1 Department of Psychology, Alma Mater Studiorum—University of Bologna, 40126 Bologna, Italy; ferdinando.toscano@unibo.it (F.T.); davide.giusino2@unibo.it (D.G.); 2 Department of Psychology, Faculty of Humanities, University of Bojnord, Bojnord 9453155111, Iran

**Keywords:** women workplace culture, WWC scale, organizational culture, female workers, Iran

## Abstract

This paper describes the validation process of the Persian version of the Women Workplace Culture Scale and provides information about the perception of this culture in an Iranian working environment. A 30-item Persian version of the Women Workplace Culture (WWC) Questionnaire was administered to women working in public departments of the city of Bojnord, Iran (N = 200). As a result of a theory- and data-driven bottom-up empirical approach, a reduced 10-item three-dimensional scale was achieved entailing (I) perceived societal barriers for career development, (II) perceived organizational barriers, and (III) sexual harassment. This parsimonious solution showed satisfactory values of reliability, factorial validity and convergent-discriminant validity analysis based on correlations with the unidimensional 10-item Perceived Stress Scale and the 12-item Career Success Questionnaire. The scale can be used to measure women workplace culture in Iran and other Persian-speaking, Islamic-Arabic countries. It can also constitute a starting point for organizational diagnosis in projects aimed to enhance working women’s occupational health and societal participation.

## 1. Introduction

The presence of women in the workforce is increasing globally [1], and it is evident that neglecting the ability of women to contribute significantly to businesses prevents society from promoting its social and economic development. The experiences of developed countries, where cultural and political changes in women’s status have provided them with greater opportunities to participate in the countries’ working life, testify the importance of women’s roles in achieving the sustainability goals of humanity [2]. Despite this evidence, however, employment data in Iran describe a scenario in which gender inequality still causes women to occupy marginal positions in the labor market, thus preventing the country from exploiting women’s talents and skills [3]. As an example, in this country, even today, university education is considered less important for girls than for boys [4]. In addition, Iran’s highly patriarchal culture not only often prevents women from holding high-level responsibilities in organizations, but also prevents managers from hiring them [5].

According to the Statistical Centre of Iran [6], the presence of women in the working population increased from 9.7% in 1956 to 13.7% in 1976, but then declined to 8.8% in 1986, fluctuating between 9% and 12% during the past two decades, and finally reaching 18% in 2019. Thus, although the situation has improved a little over the years, a steady increase has never been observed and, in general, the situation about this issue remains, in this national context, undesirable.

Despite the fact that women often show higher education levels than men [7], their current access to equal job or career opportunities and fair pay is far from being ensured. Consistent to the widely acknowledged glass ceiling phenomenon [8,9,10,11], senior management positions are especially precluded to Iranian women, thus indicating that Iranian society has failed to provide a suitable environment for the active participation of women, whose interests have been largely ignored.

### 1.1. The Role of an Organizational Culture against Women and Its Outcomes

Among the reasons for the man-woman gap worldwide, previous studies [12] have pointed out organizational culture as a major barrier to women’s difficulties to thrive in the business domain. Work and organizational psychology defines organizational culture as a framework of shared values, beliefs and norms that affect how members of an organization think and behave in relation to other people both within and outside the organization [13]. As the organizational culture influences the way members of the organization treat each other, the relationship between this construct and the way women are viewed within organizational contexts is direct, and also manifests itself when women occupy management positions and have a great potential to influence others. Not by chance, it has been shown that, due to prejudices and stereotypes related to women, their management style often tends to be framed, regardless of their abilities, in a negative way [9,10,11,12]. Women who adopt a “masculine” style of managing subordinate employees (e.g., authoritative, directive, task-oriented) are likely to be criticized for being aggressive and bossy; conversely, women who adopt a “feminine” management style (e.g., caring, empathetic, relation-oriented) are instead deemed as inefficient, ineffective or insufficient.

Such inconsistencies in the perception of the role of women lead them to fail to achieve high-quality performance standards and to experience a sense of apprehension and discomfort [9,10,11,12]. Thus, these adverse behaviors to women: (a) on the one hand, testify to the prejudice that every person can “breathe” in organizational contexts, which often transforms women themselves as promoters of this culture which, “being in the air”, they in turn incorporate; (b) on the other hand, they represent the causes of further obstacles for women, and they feed the “vicious circle” according to which women tend to perform worse than men, making the problem of the gender gap even wider.

The effects of organizational culture in sustaining the gender gap at work are manifold, and one of the main ones is its impact on organizational climate—a set of shared perceptions which are hold by organizational members regarding people attending, and events occurring within a particular organizational context or setting [14]. Due to stereotype-related negative attitudes towards women in workplaces, women’s professional knowledge, competence, skills and abilities are often called into question [12], requiring them to make extra efforts to prove their credibility [9,10,11]. Additionally, organizational culture also leads to women lacking organizational support—perceived social support from other members of the belonging organization [9,10]. Particularly, women working in patriarchal organizations reported receiving limited emotional support as well as limited access to services such as company kindergarten and day-care centers, which makes even more difficult to maintain work-life balance and coordinate multiple roles [10] when family care duties are to be fulfilled by women.

So far, the listed disadvantages experienced by women have often involved subtle aspects that are not always explicit and punishable by law or public morality. On the other hand, a further, big issue contributing to women’s feelings of unsafety and insecurity in the workplace is sexual harassment [15,16], an unpleasant and inappropriate sexually connoted behavior, which creates an upsetting and hostile work environment. Due probably to the culturally determined reluctance to disclose information about the occurrence of sexual harassment among Asian cultures [17], no accurate statistics regarding this phenomenon among working women in Iran are available. Nevertheless, studies involving hospital medical staff and hotel personnel from other Islamic-Arabic countries indicate high levels of sexual harassment episodes [18,19,20] and, therefore, also suggest that this aspect should be considered while investigating the condition of women at work in Middle Eastern contexts.

### 1.2. From Problem Framing towards Intervention

Following widely empirically tested theoretical models from the field of occupational health psychology [21], the adversities that women are currently facing in the workplace can be conceived in terms of both the presence of job demands (e.g., prejudice, stereotypes, negative attributions) and lack of job resources (e.g., organizational support, acknowledgement). Job demands are physical, psychological, social or organizational aspects of the job that require physical or psychological effort from the worker. In contrast, job resources are physical, psychological, social or organizational aspects of the job that workers can use to counterbalance costs in terms of physical, cognitive and emotional energy. Examples of job resources are cognitive and behavioral patterns (i.e., psychological resources), support from colleagues and supervisors (i.e., social resources), role clarity, job control, pay, job security and career opportunities (i.e., organizational resources). When job demands exceed job resources (i.e., a mismatch occurs between job demands and job resources), they can lead to undesirable outcomes such as job stress, work demotivation and work disengagement. For example, gender inequality has been shown to impact women’s psychological and physical stress and mental health [22]. Specifically, burnout, a syndrome that is caused by chronic work-related stress, has been recently found among female Iranian teachers [23]. Therefore, it is not to be excluded that the situation women are experiencing in workplaces due to adverse organizational cultures, and particularly in Asian and Iranian ones, may induce symptoms of work-related stress or other workplace-related mental health conditions such as work-related anxiety and depression.

The depicted scenario would call for the greater implementation of actions, strategies, initiatives and interventions aimed to promote women’s positive participation in workplaces by managing and changing the overarching organizational culture. These initiatives should follow a thorough and analytical assessment of the idiosyncratic culture characterizing one specific place of work, taking advantage of instruments and tools to sensitively operationalize and measure the wide range of experiences that women are living in the workplace nowadays.

However, traditional questionnaires are criticized for not taking into account the most recent developments that have occurred in the workplace worldwide, such as an increased presence of women. As such, they might not adequately capture the peculiar needs and psychosocial working conditions that women are currently dealing with [24], and they may be more suitable for being answered by men. When looking at the academic literature, no comprehensive questionnaire encompassing all the relevant dimensions of organizational culture that impact women’s working life seems to exist, with the exception of the Women Workplace Culture (WWC) Questionnaire, which is, to our knowledge, the only one emphasizing the barriers that women encounter within the organizational social environment.

### 1.3. The Women Workplace Culture (WWC) Questionnaire

Developed in 2002 by Bergman and Hallberg, the WWC Questionnaire derives from a grounded theory-based investigation of white women’s experiences of norms and expectations within a male-dominated industry [24]. The original version of the questionnaire is composed of three dominant factors: namely, (I) perceived burdens on me, (II) perceived burdens on women, (III) sexual harassment. All alpha coefficients were >0.70 for these three factors. A fourth factor, (IV) organizational support, also showed modest reliability (α = 0.65). Finally, a five-factor solution entailing (V) the influence of parents and siblings, was also supported by a graphic scree-test. The perceived burdens on me factor refers to perceptions of the existence of negative views and behaviors towards the single respondent female worker in her workplace. Perceived burdens on women comprises perceptions of existing negative views and behaviors towards female workers in a single workplace. The organizational support dimension corresponds to perceptions of the availability of social support towards women from other members of the organization. Finally, the influence of parents and siblings refers to the quality of female workers’ relationships with parents and siblings. In the study by [24], factors I, II and IV statistically significantly correlated with women’s ill-health, distress and job satisfaction.

Subsequently, the WWC Questionnaire validation study by [25] including N = 446, found a 24-item four-factor structure composed of perceived burdens on women (α = 0.87), personally experienced burdens (α = 0.84), sexual harassment (α = 0.80) and inadequate organizational support (α = 0.71). These factors statistically significantly correlated with self-reported ill-health, psychological distress, and work satisfaction; however, they nevertheless showed differences compared to the previous factoring found in [24], which was never tested through confirmatory factorial analysis.

Although it had some issues related to the methodology used in its development, the scale of [24] represents an element of absolute interest for studying women’s condition in working contexts. Considering a Middle Eastern context, the present study aims to perform an empirical validation of the WWC Questionnaire’s psychometric properties in the Persian language, based on the answers of a sample of Iranian female workers. To the best of the authors’ knowledge, no previous investigation of WWC validity and reliability has been conducted in the Iranian society, nor other Islamic-Arabic countries. Thus, from a psychometric point of view, the present study enriches knowledge, of the WWC Questionnaire and in particular of its validation in the Persian language, carried out in the present study. Exploiting the processes of the statistical analysis carried out, it widens the scientific literature on the theme of organizational culture towards women, providing data about women workplace culture collected within a real sample of Iranian women, whose answers we reported, for each item, at an aggregate level. In addition, it offers a tool that can be used by the scientists and professionals of those organizations which may wish to evaluate cultural opinions on the role of women in the workplace in Iran as well as in other Arab-Islamic countries with the aim, if possible, to pursue the best possible balance in terms of treatment and possibilities in the workplace between men and women.

## 2. Materials and Methods 

### 2.1. Research Design and Sample

We divided the present cross-sectional research in Study 1 and Study 2. In Study 1, we carried out a first attempt to confirm the WWC Questionnaire’s original factorial structure [24,25]. However, as the principal component analysis (PCA) did not yield fully satisfying results, we decided to perform Study 2 in order to achieve a more fitting solution.

Both Study 1 and Study 2 deployed the same recruited sample composed of 200 out of the 350 female employees working at public departments of the city of Bojnord, Iran. Data were gathered in 2017. To recruit research participants, we followed a step-sampling procedure. The minimum sample size was established based on [26,27], which suggested having five to 10 people per scale item. This decision was endorsed by [28], indicating 186 people as a sufficient sample to represent a statistical population composed of 360 people. Moreover, an analysis run in the G*Power software indicated the need for 189 respondents (α = 0.84, statistical power = 0.95) to achieve a medium effect size based on the rule of thumb by [29].

### 2.2. Procedure and Ethical Considerations

The original English version of the WWC Questionnaire [24] was translated into Persian by two Persian- and English-speaking independent translators. Then, the Persian items were transmitted to an English- and Persian-speaking third party who translated them back into English (i.e., back-translation) to prove the correctness and consistency of the Persian version. Since neither the original study by [24] nor the validation study by [25] provided unequivocal results, we decided to use all of the 30 initially available items.

Prior to the administration, the nature, scope and aim of the research were plainly explained in the language intelligible to participants. Furthermore, we clearly stated the principles of confidentiality, privacy, anonymity, voluntary participation and the right to withdraw from the study at any time without negative consequences. In the context of the present research, stressing the guarantee of anonymity was deemed particularly important, as the investigated cultural matters may lead to the occurrence of social desirability phenomena [30], threatening the validity and reliability of the achieved results. Written informed consent was obtained from each participant and all the recruited participants received the enveloped paper questionnaire and returned it to the researchers as soon as they had filled it out.

The Ethical Review Committee of the University of Bojnord, Iran, formally approved the present research.

### 2.3. Measures

The 30-item Persian version of the WWC Questionnaire was then administered to the 200 recruited Iranian working women. Respondents had to answer on Likert-type scales [30] mostly. However, the scoring method was not the same across all the WWC Questionnaire items. Thirteen items (1, 3, 4, 5, 11, 14, 15, 16, 17, 18, 23, 25, 27) require answers on a four-point scale ranging from 1 = “often” to 4 = “never”. Item 24 (“Relationships with the members of the family a person grows up with can vary a lot. Concerning your own parents and siblings please rate the importance they have had for you”) also requires answers on a 4-point scale, but the latter entails categorical answers, such as 1 = “all members (father, mother, siblings)”, 2 = “only my mother”, 3 = “only my father” and 4 = “only my siblings”. Twelve items (6, 7, 8, 10, 12, 13, 19, 20, 21, 22, 26, 28) require answers on a 3-point scale ranging from 1 = “definitely” to 3 = “not at all”. Two items (2, 9) require answers on a 5-point scale ranging from 1 = “fewer than I would wish” to 5 = “much more frequent”. Two items (29, 30) require answers on a 2-point categorical scale entailing “yes” or “no”. Items 22, 23 and 29 are reversed. Except for items 22, 23, 24 and 29, lower scores on WWC questionnaire items reflect women’s perception of the negative attitudes towards them and experienced in the workplace.

Moreover, two additional scales were administered in order to assess convergent-discriminant validity, such as the unidimensional 10-item Perceived Stress Scale (PSS) by [31], and the 12-item Career Success Questionnaire (CSQ) developed by [32] based on [33,34,35,36]. PSS measures general stress as perceived by the respondent in the last month, entails four reverse items, and presents respondents with a 5-point Likert-type scale ranging from 0 = “never” to 4 = “very often”. In the present study, we found α = 0.82 for PSS, which is similar to recent studies that have widely evaluated the reliability of this scale [37,38]. The CSQ includes six items measuring objective career success indicators (i.e., salary, promotions, benefits, occupational level; α = 0.89 in the original study by [32]) and six items measuring subjective career success indicators (i.e., improvement, security, growth, freedom, balance; α = 0.81 in the original study by [32]). In the present study, we found α = 0.93 for the whole CSQ scale.

Finally, the administered survey asked for additional demographic and job-related information, such as age, tenure, marital status, number of children, level of education and employment status.

## 3. Results

This section describes the results achieved both in Study 1 and the subsequent Study 2.

### 3.1. Study 1

One hundred and eighty participants filled out the questionnaire so that it could yield valid analyzable data. We used the IBM SPSS v26 and the IBM SPSS Amos v26 software to run statistical analyzes. Descriptive statistics were computed for both participants’ characteristics (Table 1) and WWC questionnaire items (Table 2).

The study participants were 180 women working in 16 different public departments of the municipality of Bojnord, Iran. The minimum age was 22, whereas the maximum was 53 (M = 34.90, SD = 5.88). Tenure ranged from 1 to 27 years (M = 10.16, SD = 5.63). As shown in Table 1, all respondents held a university-level educational degree. Number of children, marital status and employment status are also shown in Table 1.

Among the questions with 4-point answers, the most represented scale of response in the WWC Scale, items 23 and 27 showed the largest average scores (M = 3.16 and M = 3.59, respectively). Item 23 tackles the importance of relationships with parents and siblings, while item 27 refers to unwanted body contact or suggestions in the workplace. Also considering items 22 and 28, which refer to similar experiences but with a different reference and a different response scale, the results to these items, for the mechanisms of the construction of this instrument, show that the most positive results emerged concerned these two areas, for which women felt calmer than in others.

On the other hand, many items suggest a rather negative situation for women who, given the low scores on their responses, seemed to be suffering from an environment quite consistent with that framed by the scientific literature.

Considering that before computing means, items 22, 23, and 29 were reversed, the average WWC score was 61.79 ± 9.36 (range = 42–91, median = 61), while the modal value was 57, out of a maximum of 102 (items 24 was not included in these calculations due to its categorical response scale). Considering that, in this scale, low scores underline negative results for women, the average and modal values were fairly low scores, and, therefore, did not indicate a particularly positive situation experienced by women.

The PCA was performed with Varimax rotation, and the number of factors to be extracted was set at five, as in the original WWC model [24,25]. Item 24 was not included in this analysis due to its categorical response scale. On this basis, confirmatory factor analyzes (CFA) were performed to assess the factorial structure of the Persian version of the WWC Questionnaire.

Sampling adequacy [39,40] was supported by the Kaiser-Meyer-Olkin measure = 0.72 and Bartlett’s test of sphericity (χ^2^ = 1760.97, df = 406, *p* < 0.01). Beyond the extraction command set at five factors, the scree plot supported the adequacy of a five-factor solution, accounting for 46.37% of the explained variance, as shown in Table 3.

The extracted five-factor solution is similar to those extracted by [24,25]. To this regard, Table 4 compares the factorial solutions of this study with those of the above-mentioned research.

Subsequently, the performed CFAs showed a better fit for the extracted five-factor solution ($\chi^{2}$(df = 314) = 615.19, *p* < 0.001, RMSEA = 0.07, SRMR = 0.08, CFI = 0.79, TLI = 0.76) than for a one-factor solution ($\chi^{2}$(df = 324) = 1062.67, *p* < 0.001, RMSEA = 0.11, SRMR = 0.10, CFI = 0.48, GFI = 0.44).

The extracted five-factor solution showed a partially acceptable fit, with values of $\chi^{2}$, degrees of freedom, RMSEA and SRMR being more than acceptable according to the conventionally set cut-offs [41]. However, several reasons, both theoretical and statistical, made us hesitate about this factorial solution. First, the CFI and TLI values produced by CFA were far below the acceptable cut-offs [41], although this might be explained since the RMSEA of the null model was lower than 0.158 [42] and there were low correlations between some of the factors (for instance, the correlations between factor 5 and the other ones were, in each case, not significant). Second, some of the factors extracted by PCA mixed items referring to the individual woman’s conditions and items referring to other people or the context in general (e.g., items that ask about the specific condition of the respondent load in the same factor of “contextual” items, instead referring to the “general” situation), which is not always conceptually sound. Third, based on reliability analyzes, some factors were found to show reliability values below the desirable 0.70 [41] (factor 2 showed α = 0.60, while factor 4 and factor 5 both showed α = 0.62). Based on the above, a second study was deemed necessary to achieve a possibly more parsimonious but stronger version of the Persian WWC Questionnaire.

### 3.2. Study 2

In Study 2, we aimed to achieve a more conceptually rigorous and psychometrically valid and reliable version of the Persian WWC Questionnaire than the one achieved in Study 1. To this end, we adopted a bottom-up empirical screening approach by letting theory and data themselves drive us.

First of all, we started by collectively re-analyzing the questionnaire items’ wording in order to reach a consensus and decide how to best review the original composition of the WWC Questionnaire.

Based on the definition of organizational climate and culture [14], we kept in mind that these constructs reflected shared perceptions of individuals within a group, organization or community, rather than attitudes related to the condition of single persons. Therefore, we agreed upon dropping items 2, 4, 6, 8, 10, 12, 14, 16, 18, 20, 21, 23, 24, 26, 28, 29 and 30 from the original version of the WWC Questionnaire as they refer to individual perceptions rather than to shared beliefs aimed at describing a situation common to a group. To this regard, by carefully reading the two articles where the WWC Scale was originally taken from [24,25], no explicit indications of the reasons why the scale authors chose to include the items referring to individual perceptions, although organizational culture is a primarily socially construed phenomenon, were retrievable. A possible explanation can see this double reference as a “control”, aimed at allowing comparisons between values that refer to a personal, and therefore more difficult to declare, with a less emotionally demanding context such as the collective one and, thus, able to provide estimates of the “truthfulness” of the collected answers. However, this possible explanation is to be understood as only hypothetical.

Also, we decided to drop item 22 as it does not directly show relevance for the workplace, which is the psychosocial setting we were interested in.

Three main or macro themes emerged out of the remaining items’ contents. First, items 1, 3, 11, 15 and 17 all appeared to refer to women’s career development issues, such as inequality of professional development opportunities between men and women, unfairness of judgements on women’s work performance as compared to men, discrepancy in employment security between women and men, women’s need to put in greater efforts than men in order to get promotions, and the difference between men’s and women’s assertiveness when it comes to asking for fair compensation, promotion or professional development opportunities. Second, items 5, 7, 9 and 13 pointed out to organizational barriers, such as lack of organizational support and trust towards women, the presence of negative attitudes towards women in the working life, women’s difficulties in showing their own true self at work, and lack of attention towards women in the workplace. Third, we grouped item 25 and item 27 into a single factor addressing sexual harassment behaviors in the workplace.

On this basis, we hypothesized a three-factor structure shown in Table 5 and composed of (I) perceived societal barriers for career development, (II) perceived organizational barriers, and (III) sexual harassment.

Thus, the second part of Study 2 was aimed to test the hypothesized three-factor structure and subsequently verify whether we were able to come up with a Persian WWC questionnaire—short version proposition with good psychometric properties.

A CFA run with the hypothesized model yielded fairly acceptable values ($\chi^{2}$(df = 41) = 81.54, *p* < 0.001, RMSEA = 0.07, SRMR = 0.06, CFI = 0.92, TLI = 0.89). As shown in Table 6, the CFA showed each item loading in its hypothesized factor with values above the 0.40 cut-off [41], except for item 9 which loaded in Factor 2 with a value of 0.39. Although this value was very close to the cut-off, we decided to remove this item, and to interpret the cut-off literally, without admitting any exception.

By dropping item 9 from the hypothesized model, the CFA model results improved ($\chi^{2}$(df = 32) = 61.35, *p* < 0.001, RMSEA = 0.07, SRMR = 0.05, CFI = 0.94, TLI = 0.91) and all the item loadings were above 0.46, as shown in Table 7.

This further solution was found to be better than either a one-factor solution ($\chi^{2}$(df = 35) = 130.94, *p* < 0.001, RMSEA = 0.12, SRMR = 0.09, CFI = 0.79, TLI = 0.73) or a model with Factor 1 and Factor 2 merged and items 25 and 27 saturating into separated factors ($\chi^{2}$(df = 34) = 73.43, *p* < 0.001, RMSEA = 0.08, SRMR = 0.06, CFI = 0.91, TLI = 0.89).

On this basis, we decided to accept the three-factor structure shown in Table 8 as definitive.

Once we ascertained the good factorial structure of the solution tested, we then proceeded to examine in detail the reliability of the new scale. We found Cronbach’s [43] α = 0.72 for Factor 1, α = 0.68 for Factor 2, α = 0.70 for Factor 3, and α = 0.79 for the whole scale. The composite reliability was instead 0.77 for Factor 1, 0.71 for Factor 2, and 0.73 for Factor 3, suggesting acceptable reliability values for the reduced version of the Persian WWC Questionnaire.

Finally, by deepening the study of the validity of the scale, the Pearson’s correlations shown in Table 9 between the reduced version of the Persian WWC Questionnaire and PSS and CSQ showed statistically significant values and relationship directions that let us assume acceptable convergent-discriminant validity of the achieved solution.

## 4. Discussion

In the present research, we aimed to psychometrically validate the Women Workplace Culture Scale into Persian language. Several factorial solutions resulted from the processes we carried out. First, a five-factor model was found. This model replicated the structure deriving from the original WWC validation studies [24,25]. However, both statistical and theoretical reasons accounted for this solution not being fully satisfactory. From a statistical viewpoint, the CFAs and reliability analysis produced values that were considerably below the commonly acceptable cut-offs [39], especially referring to CFI and TLI. Furthermore, from a conceptual perspective, PCA extracted factors displaying internal inconsistencies in terms of items’ contents and meanings.

To overcome these issues and reach a fully satisfactory version of the Persian WWC Questionnaire, we used a theory- and data-driven approach for modifying the previously achieved model. This operation led us to a final, 10-item version of the Persian WWC Scale composed of three dimensions, namely (I) perceived societal barriers for career development, (II) perceived organizational barriers, and (III) sexual harassment. Dimension (I), perceived societal barriers for career development, is composed of five items, items 1, 3, 11, 15, and 17 from the original WWC validation studies [24,25], and refers to women’s career development issues, such as the inequality of professional development opportunities between men and women, unfairness of judgements on women’s work performance as compared to men, discrepancy in employment security between women and men, women’s need to put in greater efforts than men in order to get promotions, and the difference between men’s and women’s assertiveness when it comes to asking for fair compensation, promotion or professional development opportunities. Dimension (II), perceived organizational barriers, is composed of three items, namely items 5, 7 and 13 from the original WWC validation studies [24,25], and entails the organizational barriers faced by working women, such as lack of organizational support and trust towards women, the presence of negative attitudes towards women in the working life, women’s difficulties in showing their own true self at work, and lack of attention towards women in the workplace. Dimension (III), sexual harassment, is composed of two items, namely, items 25 and 27 from the original WWC validation studies [24,25] and addresses sexual harassment behaviors in the workplace. This version of the Persian WWC Scale shows a satisfactory factorial validity and reliability values, as well as good convergent-discriminant validity as compared to the unidimensional 10-item Perceived Stress Scale (PSS) by [31], and the 12-item Career Success Questionnaire (CSQ) developed by [32] based on [33,34,35,36]. Moreover, as a reduced and shorter version than both the original WWC Scale [24,25] and the Persian version of the scale achieved in Study 1, it offers a simpler and more parsimonious solution, which appears to work best according to the peculiarities of the deployed sample.

In addition to the validation of the Persian version of the scale, the second aim of this study was to disseminate the aggregate results obtained for the WWC scale that 180 women working in an Iranian public institution have on the female working culture insistent in their working context. Adding to what was already reported in the results of Study 1, the average value resulting from the short scale version we have validated in this study is 20.78, with a possible maximum of 38, we highlight how the context we have considered, while not suggesting a totally critical situation, shows how women experience a culture that is averagely averse to them in their workplace.

On the basis on these first results, we believe that further research should shed more light on the fact that today, in Iran, women still experience a culture that is contrary to their commitment as workers. We believe that the provision of a measurement tool such as the one validated here can help to pursue the objective of making work contexts, such as the one analyzed in this study, more “women-friendly”.

Some limitations in the present study should be recognized. First, as a self-report questionnaire, the WWC Scale carries with it all the typical limitations inherent to self-report instruments related to the subjectivity of responses [44]. Secondly, the generalizability of the results beyond the analyzed context as well as the Persian-speaking populations remains questionable. Third, the scale response of the questionnaire may result debatably, and for this reason, an equal response scale for all items could be a solution to be tested in the near future. Fourth, a larger sample might be used in upcoming research. Finally, we have only carried out a quantitative analysis, while some qualitative analyzes, perhaps triangulating with interviews administered to Iranian working women, could provide insights into the cultural context investigated in this research which, without reserve, must necessarily be deepened.

On the other hand, our study holds both theoretical and practical implications, especially with regard to the purpose of validating the WWC Questionnaire. On the theoretical level, the present study advances WWC state of research since, to the best of our knowledge, a confirmatory factor analysis had never been performed to investigate the five-factor structure deriving from the WWC original and validation studies [24,25]. Although we obtained similar results from the principal component analysis than those obtained from the original WWC validation studies [24,25], the current unavailability of other CFA-based validations than the one shown in the present paper might still suggest the existence of model fit issues in the original version of the questionnaire. As a consequence, it is not to be excluded that the solution we achieved in the presented study might solve potential psychometric issues in other languages versions (e.g., English) of the WWC Scale. Possibly, the achieved three-dimensional structure is capable of solving hidden and atavistic issues of a scale that has been provided with little validation both in its original form and over time. We see the investigation of these issues as a fruitful avenue for future research aiming to contribute to theory even more substantially. Interestingly, the three conceptualized dimensions, namely (I) perceived societal barriers for career development, (II) perceived organizational barriers, and (III) sexual harassment, are conceivable as examples of the job demands concepts encompassed by the widely accredited theoretical models from the field of occupational health psychology [21] which have been previously referred to in the introductory paragraph of the current paper. Furthermore, the three dimensions highlight the challenges that working women in Iran have to face, which are consistent with those reported in the available literature [8,9,10,11,12,15,16,17,18,19,20]. In addition, the present study contributes to conceptually widening the nomological horizon of the WWC construct by correlating it with two other variables, perceived stress and career success.

From an applications point of view, the 10-item Persian WWC Scale could be used as an instrument to thoroughly assess cultural representations of women in a given workplace, which would constitute the first analytical step to subsequently implementing actions, strategies, initiatives and interventions aimed to promote women’s positive participation in workplaces by managing and changing the overarching organizational culture. Particularly, organizational scientists and practitioners might use it to operationalize, measure and evaluate a range of experiences that women living and working in Iran as well as in other Islamic-Arabic countries face nowadays, thus contributing, where possible, to the elimination of the problems caused by the still existing gender gap. This scale might be especially needed in such a context where society has failed to provide a suitable environment for the active participation of women, whose motives, interests and needs have been abundantly ignored [7,8]. In particular, the use of this scale, supported by the validation of the short 10-item version, may lead to more initiatives aimed at improving women’s working conditions in terms of wellbeing at work [45], but also at exploiting the benefits of women’s inclusion in the workforce and women’s ability to contribute to business, thus, guiding the advancement of social and economic development in the contexts that will also be investigated through this tool.

## 5. Conclusions

To the best of the authors’ knowledge, no previous investigation of WWC validity and reliability has been conducted in Iranian society, nor in other Islamic-Arabic countries. This constitutes an element of novelty and innovativeness of the present research.

The 10-item Persian version of the WWC Scale has satisfactory characteristics, both theoretical and statistical. Therefore, it can be used, as in this same research, to “give a number” to the workplace culture of women in Iran and other Persian and Arab-Islamic speaking countries, following the Persian formulation we obtained and report in Appendix A. The English translation is also reported in Appendix B.

## Figures and Tables

**Table 1:** Sample demographics and job-related information.

Variable	n	%
*Marital status*		
Single	34	18.9
Married	146	81.1
*No. of children*		
<3	167	92.8
>3	13	7.2
*Education*		
Bachelor	101	56.1
Master	77	42.8
PhD	2	1.1
*Employment*		
Open-ended	56	31.1
Fixed term	89	47.2
Contract worker (staff leasing)	39	21.7

**Table 2:** Descriptive statistics for Women Workplace Culture (WWC) Questionnaire items.

Item	Scale Points	M	SD	Skewness
1. Do you think that women have fewer opportunities than men for professional development at a workplace?	4	1.76	0.91	1.08
2. Estimate your own opportunities for professional development.	5	2.39	1.29	0.74
3. Do you think that women receive more unfair judgements of their work performance than men?	4	1.77	0.85	0.10
4. How does it apply to your situation?	4	1.92	0.83	0.57
5. Do you think that men receive more organizational support and trust than women?	4	1.59	0.77	1.14
6. For you personally, would you have liked to have received…	3	1.17	0.40	2.31
7. In general terms, do you think working life is characterized by a negative attitude towards women?	3	1.93	0.53	−0.08
8. In your situation: Do you believe that the way you have been addressed at work by management and superiors has been influenced by a negative attitude towards you because you are a woman?	3	2.39	0.61	−0.48
9. Do you think it is more difficult for women than men to “be themselves” at work?	5	3.34	1.37	−0.19
10. How does it apply to your situation at work?	3	2.38	0.64	−0.56
11. Do you think that men have greater employment security than women?	4	1.73	0.94	1.01
12. How secure do you feel in your professional position?	3	2.12	0.76	−0.12
13. Do you think that women’s contributions are perceived differently, that is, do men fail to pay attention to what women say at meetings?	3	1.95	0.49	−0.13
14. How does it apply to you at work?	4	2.34	0.91	0.04
15. Do you think that women have to be more accomplished in their work than men in order to be promoted?	4	1.74	0.83	0.98
16. How does it apply to your situation?	4	1.84	0.92	0.88
17. Do you think that women are less assertive compared to men to obtain fair compensation, promotion or opportunities for professional development?	4	1.88	0.89	0.71
18. How does it apply to your situation?	4	1.79	0.80	0.87
19. Do you think that women receive enough organizational support in order to manage their professional work and their domestic responsibilities?	3	1.47	0.57	0.74
20. How does it apply to you?	3	1.62	0.68	0.63
21. If you have a partner, do you receive sufficient support from your partner?	3	2.38	0.62	−0.46
22. About women’s ability to manage difficulties that arise, how important do you think a person’s relationships to parents and siblings are?	3	2.74	0.50	−1.80
23. How does it apply to your situation?	4	3.16	0.72	−0.61
24. Relationships with the members of the family a person grows up with can vary a lot. Concerning your own parents and siblings please rate the importance they have had for you.	4	1.33	0.67	2.33
25. Do unwelcome sexual connotations glances, gestures, or comments occur at your place of work?	4	2.83	1.06	−0.20
26. Has any of the above happened to you personally?	3	2.18	0.76	−0.31
27. Does unwelcome conscious body contact or unwelcome suggestions occur at your place of work?	4	3.59	0.78	−1.89
28. Has any of the above happened to you personally?	3	2.73	0.57	−1.99
29. Generally speaking, if you experience a particular difficulty: Have you somewhere or somebody to speak openly about it with?	2	1.40	0.50	0.54
30. Do you have ever thought about leaving your job because of gender-related problems?	2	1.66	0.48	−0.66

**Table 3:** Rotated component matrix resulting from principal component analysis (PCA) ^1^.

Item	Factor Loadings				
	I	II	III	IV	V
3	0.70				
15	0.70				
17	0.68				
16	0.64				
1	0.64				
18	0.63				
5	0.56				
13	0.50		0.40		
11	0.46				
7	0.42				
14	0.41				
25		0.83			
27		0.80			
28		0.77			
26		0.65			
30					
29					
10			0.66		
12			0.64		
8			0.58		
9			0.47		
4			0.43		
2			0.42		
23				0.78	
21				0.74	
22				0.60	
19					0.85
20					0.73
6					0.45
Eigenvalue	5.95	2.44	2.14	1.80	1.52
Explained variance	20.51	8.41	7.38	6.20	5.25

^1^ Only values ≥ 40 are reported.

**Table 4:** Comparison of WWC factorial solutions from the present study [24,25].

Factor	Present Study	Original Paper [24]	Validation Paper [25]
Perceived burden on women	1, 3, 5, 7, 11, 13, 14, 15, 16, 17, 18	1, 3, 5, 7, 9, 15	1, 3, 5, 7, 11, 13, 14, 15, 16, 18
Personally experienced burdens	2, 4, 8, 9, 10, 12	2, 4, 6, 8, 10, 13, 14, 16, 18	2, 4, 6, 10, 12
Sexual harassment	25, 26, 27, 28	25, 26, 27, 28	25, 26, 27, 28
Inadequate organizational support	6, 19, 20	11, 12, 19, 20	19, 20, 21
Influence by parents and siblings ^1^	21, 22, 23	22, 23, 17	22, 23

^1^ The name of the influence by parents and siblings factor was changed to influence by spouse and family in the present study. Items with factor loadings < 0.40 in the considered studies were not included in this table. References [24,25] used the same cut-off (<0.40) we used in the present study.

**Table 5:** Hypothesized Persian WWC questionnaire’s three-factor structure.

Hypothesized Factors	Items
Perceived societal barriers for career development	1, 3, 11, 15, 17
Perceived organizational barriers	5, 7, 9, 13
Sexual harassment	25, 27

**Table 6:** Factor loadings resulting from the confirmatory factor analyzes (CFA) of the hypothesized Persian WWC Questionnaire’s new three factor structure.

Items	Factor Loadings		
	I	II	III
Item 1	0.64		
Item 3	0.74		
Item 11	0.45		
Item 15	0.66		
Item 17	0.46		
Item 5		0.69	
Item 7		0.65	
Item 9		0.39	
Item 13		0.67	
Item 25			0.92
Item 27			0.61

**Table 7:** Factor loadings resulting from the confirmatory factor analyzes (CFA) of the hypothesized Persian WWC Questionnaire’s new three-factor structure after dropping item 9.

Items	Factor Loadings		
	I	II	III
Item 1	0.63		
Item 3	0.74		
Item 11	0.46		
Item 15	0.65		
Item 17	0.46		
Item 5		0.69	
Item 7		0.65	
Item 13		0.67	
Item 25			0.97
Item 27			0.58

**Table 8:** Definitive version of Persian WWC Questionnaire’s three-factor structure.

Factors	Items
Perceived societal barriers for career development	1, 3, 11, 15, 17
Perceived organizational barriers	5, 7, 13
Sexual harassment	25, 27

**Table 9:** Matrix of correlations among reduced Persian WWC, Perceived Stress Scale (PSS), and Career Success Questionnaire (CSQ).

	WWC Fact. 1	WWC Fact. 2	WWC Fact. 3	WWC Scale	PSS	CSQ
WWC Factor 1	*(0.72)*	0.61 **	0.23 **	0.89 **	−0.33 **	0.25 **
WWC Factor 2		*(0.68)*	0.34 **	0.80 **	−0.20 **	0.25 **
WWC Factor 3			*(0.71)*	0.58 **	−0.22 **	−0.05
WWC Scale				*(0.79)*	−0.34 **	0.22 **
PSS					*(0.82)*	−0.31 **
CSQ						*(0.93)*

** *p* < 0.01.

## Appendix B. English Translation of the 30-Item Persian Version of the WWC Questionnaire (WWC Short Scale Items Marked with *)

**Item**	**1**	**2**	**3**	**4**	**5**
1. Do you think that women have fewer opportunities than men for professional development at a workplace? *	Often	Sometimes	Rarely	Never	-
2. Estimate your own opportunities for professional development.	Fewer than I would wish	Sometimes less	Exactly how I would wish	Sometimes more	Much more frequent
3. Do you think that women receive more unfair judgements of their work performance than men? *	Often	Sometimes	Rarely	Never	-
4. How does it apply to your situation?	Often	Sometimes	Rarely	Never	-
5. Do you think that men receive more organizational support and trust than women? *	Often	Sometimes	Rarely	Never	-
6. For you personally, would you have liked to have received…	Definitely	To some extent	Not at all	-	-
7. In general terms, do you think working life is characterized by a negative attitude towards women? *	Definitely	To some extent	Not at all	-	-
8. In your situation: Do you believe that the way you have been addressed at work by management and superiors has been influenced by a negative attitude towards you because you are a woman?	Definitely	To some extent	Not at all	-	-
9. Do you think it is more difficult for women than men to “be themselves” at work?	Fewer than I would wish	Sometimes less	Exactly how I would wish	Sometimes more	Much more frequent
10. How does it apply to your situation at work?	Definitely	To some extent	Not at all	-	-
11. Do you think that men have greater employment security than women? *	Often	Sometimes	Rarely	Never	-
12. How secure do you feel in your professional position?	Definitely	To some extent	Not at all	-	-
13. Do you think that women’s contributions are perceived differently, that is, do men fail to pay attention to what women say at meetings? *	Definitely	To some extent	Not at all	-	-
14. How does it apply to you at work?	Often	Sometimes	Rarely	Never	-
15. Do you think that women have to be more accomplished in their work than men in order to be promoted? *	Often	Sometimes	Rarely	Never	-
16. How does it apply to your situation?	Often	Sometimes	Rarely	Never	-
17. Do you think that women are less assertive compared to men to obtain fair compensation, promotion or opportunities for professional development? *	Often	Sometimes	Rarely	Never	-
18. How does it apply to your situation?	Often	Sometimes	Rarely	Never	-
19. Do you think that women receive enough organizational support in order to manage their professional work and their domestic responsibilities?	Definitely	To some extent	Not at all	-	-
20. How does it apply to you?	Definitely	To some extent	Not at all	-	-
21. If you have a partner, do you receive sufficient support from your partner?	Definitely	To some extent	Not at all	-	-
22. About women’s ability to manage difficulties that arise, how important do you think a person’s relationships to parents and siblings are?	Definitely	To some extent	Not at all	-	-
23. How does it apply to your situation?	Often	Sometimes	Rarely	Never	-
24. Relationships with the members of the family a person grows up with can vary a lot. Concerning your own parents and siblings please rate the importance they have had for you.	All members (father, mother, siblings)	Only my mother	Only my father	Only my siblings	-
25. Do unwelcome sexual connotations glances, gestures, or comments occur at your place of work? *	Often	Sometimes	Rarely	Never	-
26. Has any of the above happened to you personally?	Definitely	To some extent	Not at all	-	-
27. Does unwelcome conscious body contact or unwelcome suggestions occur at your place of work? *	Often	Sometimes	Rarely	Never	-
28. Has any of the above happened to you personally?	Definitely	To some extent	Not at all	-	-
29. Generally speaking, if you experience a particular difficulty: Have you somewhere or somebody to speak openly about it with?	Yes	No			
30. Do you have ever thought about leaving your job because of gender-related problems?	Yes	No			
